# Acute neuro-endocrine profile and prediction of outcome after severe brain injury

**DOI:** 10.1186/1757-7241-21-33

**Published:** 2013-04-20

**Authors:** Zandra Olivecrona, Per Dahlqvist, Lars-Owe D Koskinen

**Affiliations:** 1 Department of Pharmacology and Clinical Neuroscience, Division of Neurosurgery, Umeå University, SE 90185, Umeå, Sweden; 2 Department of Public Health and Clinical Medicine, Umeå University, SE 90185, Umeå, Sweden

**Keywords:** Severe traumatic brain injury, Hypopituitarism, Outcome, ICP targeted therapy, Hypothalamic-pituitary dysfunction, Prostacyclin

## Abstract

**Object:**

The aim of the study was to evaluate the early changes in pituitary hormone levels after severe traumatic brain injury (sTBI) and compare hormone levels to basic neuro-intensive care data, a systematic scoring of the CT-findings and to evaluate whether hormone changes are related to outcome.

**Methods:**

Prospective study, including consecutive patients, 15–70 years, with sTBI, Glasgow Coma Scale (GCS) score ≤ 8, initial cerebral perfusion pressure > 10 mm Hg, and arrival to our level one trauma university hospital within 24 hours after head trauma (n = 48). Serum samples were collected in the morning (08–10 am) day 1 and day 4 after sTBI for analysis of cortisol, growth hormone (GH), prolactin, insulin-like growth factor 1 (IGF-1), thyroid-stimulating hormone (TSH), free triiodothyronine (fT3), free thyroxine (fT4), follicular stimulating hormone (FSH), luteinizing hormone (LH), testosterone and sex hormone-binding globulin (SHBG) (men). Serum for cortisol and GH was also obtained in the evening (17–19 pm) at day 1 and day 4. The first CT of the brain was classified according to Marshall. Independent staff evaluated outcome at 3 months using GOS-E.

**Results:**

Profound changes were found for most pituitary-dependent hormones in the acute phase after sTBI, i.e. low levels of thyroid hormones, strong suppression of the pituitary-gonadal axis and increased levels of prolactin. The main findings of this study were: 1) A large proportion (54% day 1 and 70% day 4) of the patients showed morning s-cortisol levels below the proposed cut-off levels for critical illness related corticosteroid insufficiency (CIRCI), i.e. <276 nmol/L (=10 ug/dL), 2) Low s-cortisol was not associated with higher mortality or worse outcome at 3 months, 3) There was a significant association between early (day 1) and strong suppression of the pituitary-gonadal axis and improved survival and favorable functional outcome 3 months after sTBI, 4) Significantly lower levels of fT3 and TSH at day 4 in patients with a poor outcome at 3 months. 5) A higher Marshall CT score was associated with higher day 1 LH/FSH- and lower day 4 TSH levels 6) In general no significant correlation between GCS, ICP or CPP and hormone levels were detected. Only ICP_max_ and LH day 1 in men was significantly correlated.

**Conclusion:**

Profound dynamic changes in hormone levels are found in the acute phase of sTBI. This is consistent with previous findings in different groups of critically ill patients, most of which are likely to be attributed to physiological adaptation to acute illness. Low cortisol levels were a common finding, and not associated with unfavorable outcome. A retained ability to a dynamic hormonal response, i.e. fast and strong suppression of the pituitary-gonadal axis (day 1) and ability to restore activity in the pituitary-thyroid axis (day 4) was associated with less severe injury according to CT-findings and favorable outcome.

## Introduction

Traumatic brain injury (TBI) remains one of the major causes of death and disability worldwide. The pituitary is particularly vulnerable to head trauma due to the anatomical location of the gland within the sella turcica as well as its fragile infundibular hypothalamic structure and its vascular supply. Pituitary insufficiency after trauma was first reported in 1914 [1] and post-mortem evidence dating back several decades show pituitary gland infarctions in up to one-third of patients deceased shortly after TBI [2]. Injury mechanisms of hypothalamic-pituitary damage due to TBI include direct mechanical/shearing injury to the pituitary stalk and the vulnerable long hypophyseal vessels, which may result in anterior lobe infarction and secondary injuries due to increased intracranial pressure, hypotension, hypoxia and vasospasm. The posterior pituitary is less susceptible to injury due to less fragile vascular supply [3]. Nevertheless, pituitary insufficiency after TBI has until recently been considered a rare event, with sparse data derived from case reports and case series [3,4]. However, reports from recent years have suggested permanent pituitary insufficiency after traumatic head injury to be far more common than previously thought [5,6].

In the acute phase after TBI, depending on TBI severity and location an acute post-traumatic hypothalamic and pituitary tissue damage is likely to occur in many patients early after trauma, with concomitant disturbances in hormone secretion. Most important in the acute phase is not to overlook an acute insufficiency in the hypothalamus-pituitary-adrenal axis (HPA-axis) with inadequate cortisol secretion, which is a life-threatening condition and must be correctly diagnosed and promptly treated. However, solid diagnostic criteria of cortisol insufficiency in critical illness are still lacking and under debate [7,8]. Furthermore, the known roles of GH, IGF-1, estrogen and testosterone upon brain function and plasticity propose that inadequate levels after sTBI may have both acute and long-term significance upon the recovering brain [9-13]. GH and IGF-1 receptors are abundant in the brain, GH is involved in vascular reactivity, vascular tone and CNS repair processes, while IGF-1 seems to be important in re-myelination and avoidance of demyelination [11,12,14]. There is proof of that estrogen and progesterone are neuro-protective, whereas androgens have been reported both to exacerbate and protect against neuronal damage, probably in a time and dose-dependent manner [15].

Previous reports on the neuro-endocrine changes in the acute phase of moderate to severe TBI show evidence of central hypogonadism in 25–80%, thyroid hormone deficiency in 2–15%, hyperprolactinemia in more than 50%, GH deficiency in 18% and cortisol deficiency in 13%. However, most previous reports are on mixed materials, i.e. mild, moderate and severe traumatic brain injury, acute phase definition varies and the treatment given for the sTBI is not always clearly described.

At the neurosurgical department at Umeå University Hospital, all patients suffering from sTBI are treated according to an intracranial pressure (ICP) targeted protocol based on aggressive neurosurgery and the Lund concept resulting in a mortality of less than 15% [16].

The aims of this prospective study were to investigate the prevalence and dynamics of very early pituitary-related hormonal dysfunction and the relation to basic neuro-intensive care data, CT-findings and possible prognostic implications in a strictly defined group of patients with sTBI (Glasgow coma scale, GCS < 9), treated according to the Lund concept.

## Material and methods

In the northern part of Sweden, the department of neurosurgery in Umeå has a regional responsibility of about 900 000 inhabitants. The area corresponds approximately to the area of Great Britain. All referring hospitals in the catchment area of the department refer patients with sTBI. All patients treated for sTBI in the period from January 1st 2002 to December 31st 2005 were included in the study if inclusion criteria were met. The inclusion criteria were: age 15–70 years, arrival in the department within 24 hours of trauma, verified traumatic brain injury, GCS at intubation and sedation of GCS 8 or less and a first measured CPP of 10 mmHg or more. TBI severity was defined by the GCS, and based on the first score registered after resuscitation. If there were doubts about the GCS when the patients arrived from the referring hospitals a re-evaluation was done. Exclusion criteria were: pregnant or breastfeeding woman, penetrating head injury and medication with glucocorticoids.

The patients were part of a prospective randomized blinded placebo controlled study on the effect of prostacyclin in severe traumatic head injury [16].

All patients were initially sedated with midazolam and fentanyl. No patient received Etomidate. Multimodal monitoring was applied. Invasive arterial blood pressure and ICP, using an intra-parenchymal pressure-measuring device (Codman MicroSensor, Johnson & Johnson Professional Inc., Raynham, MA, USA), were continuously measured. CPP was automatically calculated. Data were digitally stored using the Picis system (Picis, Inc., Wakefield, MA, USA) and the LabPilot (CMA Microdialysis, Solna, Sweden).

Patients were treated with no head elevation and the arterial baseline level was set at the heart level. Thus, no correction for CPP was needed. The patients were mechanically ventilated (P_a_O_2_ ≥12 kPa and P_a_CO_2_ 4.5-5.5 kPa). The goal of the treatment was to maintain ICP <20 mmHg, not allowing CPP <50 mmHg. Hourly ICP and CPP were calculated by using all the minute-to-minute ICP and CPP values during the first 5 days. The hour with the highest ICP and lowest CPP was identified as ICP_max_ and CPP_min_.

The patients were kept normo-volemic. The crystalloid and colloid osmotic pressures were kept normal by infusion of red blood cells, albumin, glucose solutions and Ringer’s acetate and sodium (S-Hb >110 g/L, S-alb >40 g/L, Na^+^ ≥ 140 mmol/L). The fluid balance was kept neutral and furosemide was used when indicated. After hemodynamic stabilization, clonidine and metoprolol were given as continuous intravenous infusions, in order to normalize the blood pressure and to reduce the transcapillary hydrostatic pressure. These drugs also reduce the stress level mediated by the sympathetic nervous system. Muscle relaxants and steroids are not a part of the treatment regime and thus not used. Further, mass lesions were surgically removed. If ICP, despite of the above measures, was not brought under control, additional sedation with low-dose thiopental, placement of a ventriculostomy and uni- or bilateral hemicraniectomy with duraplasty were used.

The severity of the trauma was assessed by ISS [17] and the brain tissue damage by the Marshall classification [18].

Serum samples were collected in the morning (08–10 am) day 1 and day 4 after sTBI for analysis of cortisol, growth hormone (GH), prolactin, insulin-like growth factor 1 (IGF-1), thyroid-stimulating hormone (TSH), free triiodothyronine (fT3), free thyroxine (fT4), follicular stimulating hormone (FSH), luteinizing hormone (LH), testosterone and sex hormone-binding globulin (SHBG) (men). Serum for cortisol and GH was also obtained in the evening (17–19 pm) at day 1 and day 4. The samples were immediately centrifuged and stored at -70°C until analysis. All hormones were analyzed at the accredited clinical chemistry laboratory at Umeå university hospital. Serum cortisol, TSH, fT3, fT4, FSH, LH, prolactin and SHBG were analyzed by electrochemiluminescence immunoassay (ECLIA; Modular Analytics E170, Roche, GmbH, Hannheim, Germany). Serum testosterone was analyzed by Coat-a-count RIA (Siemens). Serum GH and IGF-1 was measured by DPC Immulite 2000 (Siemens). Calculated free testosterone (f_c_-testosterone) levels were calculated using total testosterone, SHBG and albumin levels [19]. Clinical outcome was assessed at 3 months after trauma by independent staff and performed with structured interviews according to the extended Glasgow Outcome Scale GOS-E. The clinical outcome is reported as GOS score at 3 months. GOS was also dichotomized into unfavorable (GOS 1–3)/favorable (GOS 4–5) and into deceased/alive for further outcome analysis.

Values are reported as mean ± standard error of the mean (SEM) for continuous data and for non-parametric and ordinal variables as median and range. A two-tailed Student’s t-test was applied for continuous data. Comparison of cortisol levels between groups and comparison of all hormone levels between deceased and alive was made using the non-parametric Wilcoxon sign-rank test, due to large variations in the cortisol levels and very few patient deceased at 3 months. Correlation analyses were made using Pearson test for continuous data and Spearman’s rho test when at least one parameter was ordinal. Factors influencing outcome was explored using logistic regression. Prediction of outcome was analyzed using receiver operated characteristics (ROC) curve method. The JMP (9.0.0) statistical package was used (SAS Institute Inc. USA). A p ≤ 0.05 was considered statistically significant.

The regional ethical board at Umeå University approved the study (00–175, 05-007M). The study was also approved by the Swedish Medical Products Agency (151:633/01) and the study is registered as a clinical trial (ClinicalTrial.gov identifier NCT01363583).

## Results

Out of 89 patients, 48 patients fulfilled the inclusion criteria [16]. One patient died soon after admission, thus hormone samples could not be obtained. Due to cervical spine injury two patients were treated with high-dose methylprednisolone before the transfer to the neurosurgical trauma level one unit in Umeå. This treatment was withdrawn at our department and these patients were excluded from the study. Thus our results are based on 45 patients, 15 women and 30 men, none of these received glucocorticoid treatment. Due to technical reasons one or two hormone analysis are sometimes lacking and this is denoted in the text. Mean age was 35.7 ± 2.2, (range 15–64), median ISS was 29 [9-43] and median GCS at intubation and sedation was 6 [3-8]. The trauma was caused by road accidents (car, motorbike, bicycle and pedestrian) 28/45, snow mobile accidents 4/45, falls 10/45, assaults 2/45 and sport 1/45. Patients were hospitalized at the neuro-intensive care unit for a mean of 12.5 ± 0.6 days, median 12.3 days (3.5-23.7). Two patients died during the neuro-intensive care treatment, due to therapy refractive high ICP. At 3 months mortality was 8.9% (4/45), median GOS was 4 [1-5] and favorable outcome (GOS 4–5) was found in 53.3% of the patients.

There was no significant difference regarding age, sex distribution, initial GCS, ISS or clinical outcome at 3 months between the prostacyclin and placebo treated groups [16]. There were also no significant differences in any of the measured hormone levels at day 1 or 4 after sTBI between patients treated with prostacyclin and placebo (data not shown). Therefore, the results represent the whole patient group (prostacyclin-and placebo treated groups together).

**Figure 1:**
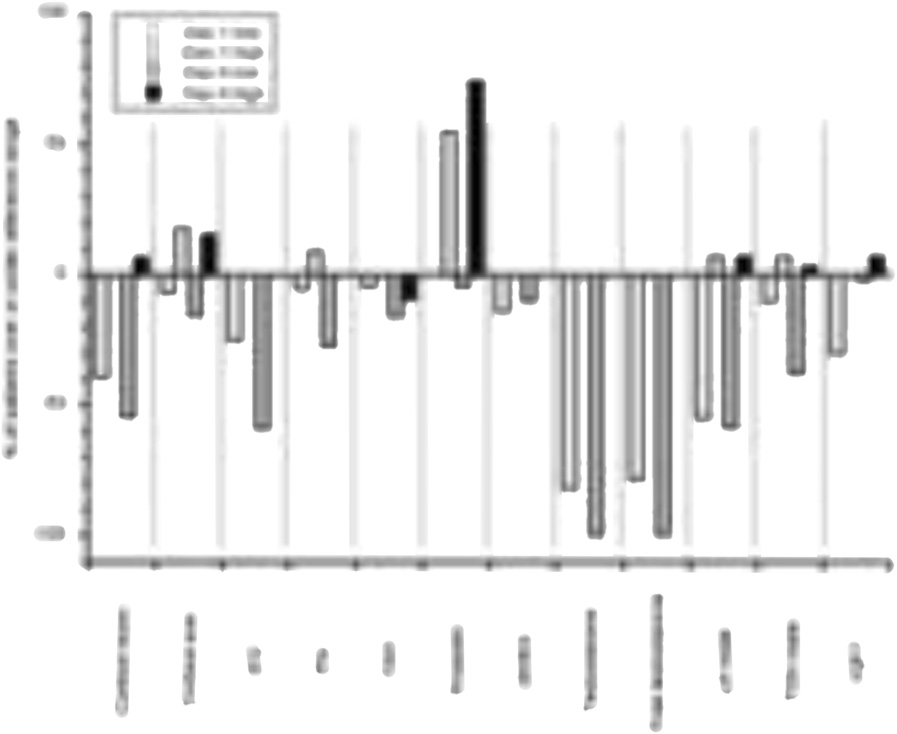
**Proportions of patients (n = 45) presenting hormone values above or below laboratory reference interval day 1 and day 4 after sTBI.** Reference intervals are given in Table 1.

Substantial effects of sTBI on hormone levels were observed. Figure 1 depicts the proportion of patients with hormone levels above or below normal reference ranges for our accredited laboratory. Large proportions of the patients showed elevated levels of prolactin and low cortisol, fT3, testosterone, LH and FSH levels.

### Hypothalamic-pituitary-adrenal axis

Mean serum cortisol day 1 after sTBI was within reference ranges with a non-significant increase from day 1 to day 4 (Table 1). However, there was a substantial individual variation. Thus, using the proposed limit for critical illness related corticosteroid insufficiency (CIRCI) [20] of total serum cortisol <276 nmol/L (10 ug/dL) day 1 cortisol was low in 24/44 (54.5%) patients in the morning and 23/44 (52.3%) in the evening. Day 4 the corresponding figures were 31/44 (70.5%) in the morning and 26/44 (59.1%) in the evening. The number of patients with very low serum cortisol (<100 nmol/L) day 1 after sTBI was 8/43 (18.6%) in the morning and 7/44 (15.9%) in the evening. Day 4, the number of patients with morning cortisol below 100 nmol/L was 10/44 (22.7%) and in the evening 11/45 (24.4%). Abnormally high morning cortisol levels were considered as >800 nmol/L and in the evening >600 nmol/L in accordance with laboratory reference range. Cortisol levels exceeding reference range day 1 after sTBI was found in 0/43 (morning) and 8/44 (18.2%, evening). The corresponding figures day 4 was 3/44 (6.8%) and 7/45 (15.6%). There was a trend towards higher morning cortisol levels day 1 in subjects deceased at 3 months (497 ± 143 nmol/L) as compared with survivors (282 ± 31 nmol/L) (p = 0.13). However, there were no statistically significant differences in cortisol levels day 1 or day 4 between deceased vs. alive subjects at 3 months or between patients with unfavorable vs. favorable outcome. No correlation was seen between cortisol and GCS, ISS, Marshall grade, GOS, ICP_max_ or CPP_min_.

**Table 1:** Hormone levels day 1 and day 4 after trauma

**Hormone**	**Reference range**	**Day 1 mean ± sem**	**Day 4 mean ± sem**	**p-value**
Cortisol morning	200-800	302 ± 30	494 ± 168	ns
(nmol/L)				
Cortisol evening	50-600	355 ± 38	610 ± 222	ns
(nmol/L)				
fT4	12-22	17.3 ± 0.6	13.8 ± 0.4	0.0001
(pmol/L)				
fT3	3.1- 6.8	3.7 ± 0.1	2.8 ± 0.1	0.0001
(pmol/L)				
TSH	0.27-4.20	0.9 ± 0.1	1.7 ± 0.3	0.03
(mlU/L)				
Prolactin males	86-324	357 ± 25	409 ± 27	0.02
(mlU/L)				
Prolactin females	102-496	571 ± 38	795 ± 75	0.02
(mlU/L)				
SHBG males	14-48	22.1 ± 1.5	24.9 ± 2.0	ns
(nmol/L)				
Testosterone males	9.4-37	4.9 ± 0.9	1.4 ± 0.2	0.002
(nmol/L)				
f_c_-Testosterone (pmol/L)	≥225	123 ± 24	31.8 ± 5.3	0.001
LH males	1.7-8.6	3.7 ± 1.0	2.7 ± 0.9	ns
(IU/L)				
FSH males	1.5-12	4.6 ± 1.3	2.9 ± 1.1	0.003
(IU/L)				
GH morning	-	16.0 ± 2.5	15.4 ± 2.8	ns
(mIU/L)				
GH evening	-	19.2 ± 3.1	13.0 ± 1.9	0.05
(mIU/L)				
IGF-1	Age dependent	126 ± 8.3	193 ± 11.1	0.0001
(μg/L)				

Values are means ± sem.

Non-significant (ns).

### Thyroid axis

Mean serum fT4 levels decreased significantly (−20.4%) from day 1 to day 4 after TBI (p < 0.0001), (Table 1). The level of fT4 was below reference range (12–22 pmol/L) in 4/44 (5.5%) of the patients at day 1 and in 12/44 (27.3%) at day 4, whereas fT4 above normal was found in 4/44 (9.1%) of the patients day 1 but none at day 4. There was no statistically significant difference in fT4 levels between the deceased and alive patients or in the unfavorable/favorable outcome groups at 3 months. No significant correlations were found between fT4 and GCS, ISS, Marshall grade, ICP_max_, CPP_min_ or GOS.

**Figure 2:**
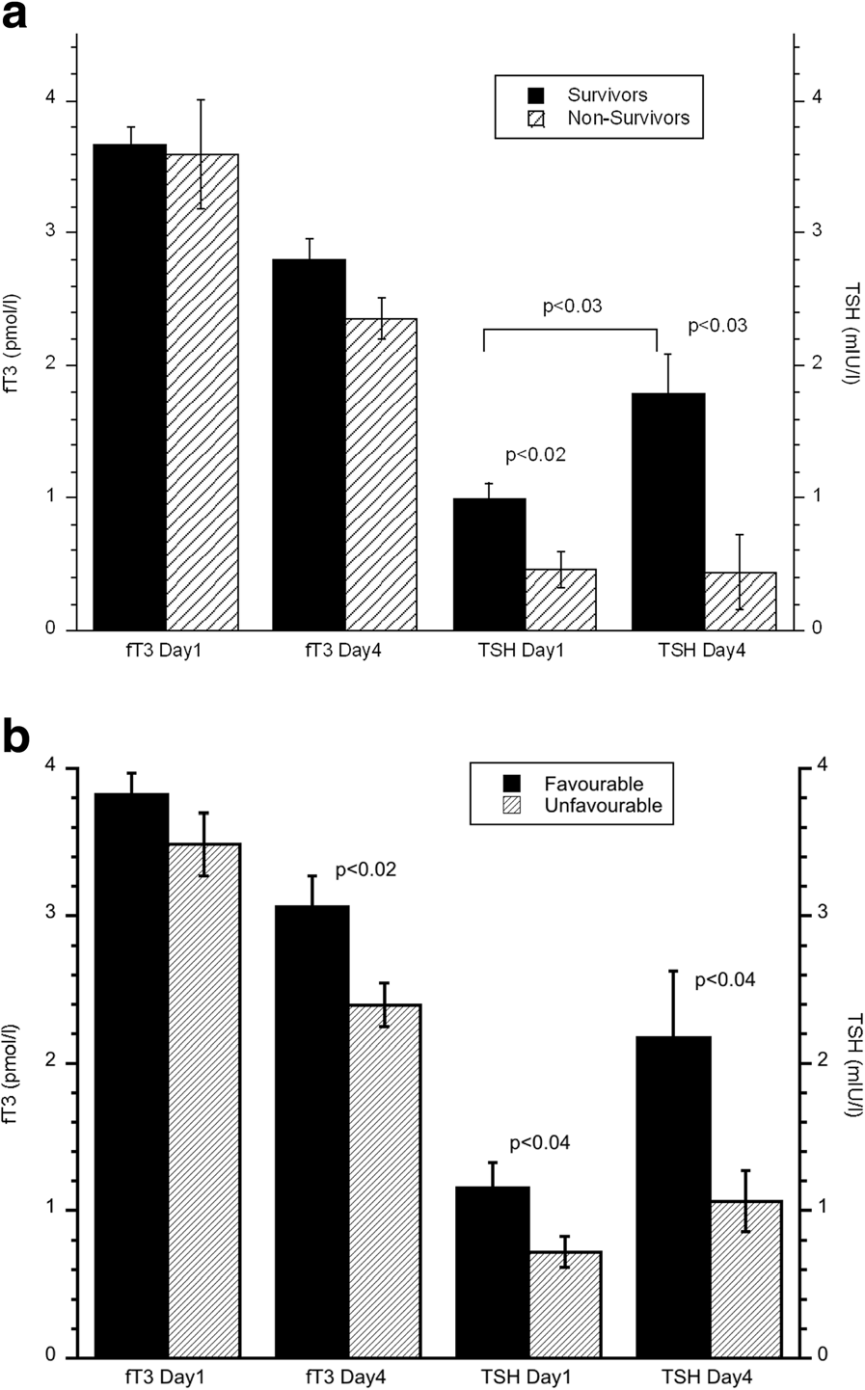
**a) Serum fT3 and TSH levels day 1 and day 4 in survivors and non-survivors 3 months after injury.** Wilcoxon sign rank test between groups and paired Student´s t-test between day 1 and day 2 results. Values are means ± sem. **b**) Serum fT3 and TSH levels day 1 and day 4 in patients with favorable (GOS 4-5) and unfavorable (GOS 1-3) outcome 3 months after injury. Wilcoxon sign rank test between groups. Values are means ± sem.

Mean fT3 levels followed fT4 and decreased significantly (−24.3%) from day 1 to day 4 (p < 0.0001), (Table 1). Serum fT3 was below reference range (3.1-6.8 pmol/L) in 11/44 (25.0%) of the patients at day 1 and in 26/44 patients (59.1%) at day 4, see Figure 1. Patients with an unfavorable outcome at 3 months (GOS 1–3) had significantly lower fT3 day 4 (2.4 ± 0.1 pmol/L) as compared to patients with favorable outcome (3.1 ± 0.2 pmol/L) (p < 0.02), see Figure 2b. There was a significant positive correlation between fT3 at day 4 and GOS at 3 months ((Spearman’s rho) ρ = 0.37, p < 0.02). There was no significant correlation between fT3 and GCS, ISS, Marshall grade, ICP_max_ or CPP_min_.

Mean serum TSH increased significantly (+89%) from day 1 to day 4 (p < 0.03), Table 1. However, TSH showed a greater variability day 4 than day 1 after sTBI. Thus, day 1 only 2/44 (4.5%) of the patients showed TSH below and none above reference values (0.27-4.2 mIU/L), whereas TSH was low in 7/44 (15.9%) and high in 4/44 (9.1%) at day 4 (Figure 1).

Acute phase TSH levels were significantly lower in patients deceased at 3 months after sTBI as compared to survivors, both at day 1 (0.5 ± 0.1 v.s 1.0 ± 0.1 mlU/L; p < 0.02) and day 4 (0.4 ± 0.3 v.s 1.8 ± 0.3 mIU/L; p < 0.03) Figure 2a. Accordingly, the significant increase of TSH from day 1 to day 4 was only found in survivors (p < 0.03) whereas TSH in non-survivors remained low. Similarly, TSH levels were significantly lower in the unfavorable group as compared to the favorable outcome group at 3 months, with day 1 TSH 0.7 ± 0.1 v.s 1.2 ± 0.2 mIU/L (p < 0.04) and day 4 TSH: 1.1 ± 0.2 mIU/L v.s 2.2 ± 0.5 mIU/L (p < 0.04) (Figure 2b). A tendency to an increase of TSH from day 1 to day 4 was only found in the favorable outcome group (p = 0.08). Day 4 serum TSH was significantly correlated to the GOS score at 3 months (ρ = 0.3, p < 0.05). Day 1 TSH was negatively correlated to Marshall grade (ρ = −0.48, p < 0.001), but not to GCS, ISS, CPP_min_, ICP_max_.

### Prolactin

Elevated levels of serum prolactin were observed in 14/29 (48.3%) of the men and in 10/15 (66.7%) of the women at day 1 after sTBI (Figure 1). At day 4 the number of male patients with supra-normal prolactin levels had increased to 21/29 (72.4%), whereas one male showed low prolactin. The corresponding results in women were 13/15 (86.7%) and 0/15. Mean prolactin levels increased from day 1 to day 4 in both men and women (p < 0.02) (Table 1). There was no statistically significant difference in the prolactin levels between deceased/alive subjects or in the unfavorable/favorable groups at 3 month. Prolactin levels were not correlated to GCS, ISS, Marshall grade, GOS, CPP_min_ and ICP_max_.

### Pituitary – gonadal axis in males

A strong suppression of the pituitary-gonadal axis was found in the acute phase after sTBI. Total testosterone levels were very low at day 1 after sTBI and decreased significantly from day 1 to day 4 (p < 0.002), see Table 1. Thus, total testosterone was below reference range in 23/28 (82.1%) at day 1 and in 29/29 (100%) at day 4 after sTBI (Figure 1). Total testosterone levels day 1 was significantly lower in survivors (4.0 ± 0.9 nmol/L) than in patients deceased at 3 months (9.9 ± 2.8 nmol/L) (p < 0.05). Total testosterone day 1 correlated negatively to GOS at 3 months (ρ = −0.39, p < 0.04), but there was no significant difference in mean serum testosterone between the unfavorable vs. favorable groups at 3 month. No correlation was found between total testosterone and GCS, ISS, Marshall grade, ICP_max_ and CPP_min_.

Mean SHBG levels were within normal levels both at day 1 and 4, see Table 1. SHBG was below reference range in 4/29 (13.8%) patients day 1 and day 4 2/29 (6.9%). No supranormal value was identified.

Free calculated testosterone levels decreased from 123.4 ± 24.0 pmol/L at day 1 to 31.8 ± 5.3 pmol/L at day 4 (p < 0.001), Table 1. The f_c_-testosterone was low (<225 pmol/L) in 22/28 (78.6%) men day 1 and in 29/29 (100%) day 4, Figure 1. Also f_c_-testosterone day 1 after TBI was significantly lower in patients alive (100.1 ± 22.5 pmol/L) compared to deceased (263.3 ± 74.1 pmol/L) 3 months after TBI (p < 0.04). This difference was not found between the unfavorable/favorable outcome groups. A negative correlation between day 1 f_c_-testosterone and GOS at 3 months approached statistical significance (ρ = −0.36, p = 0.063). There was no correlation between f_c_-testosterone levels and GCS, ISS, Marshall grade, ICP_max_ and CPP_min_.

**Figure 3:**
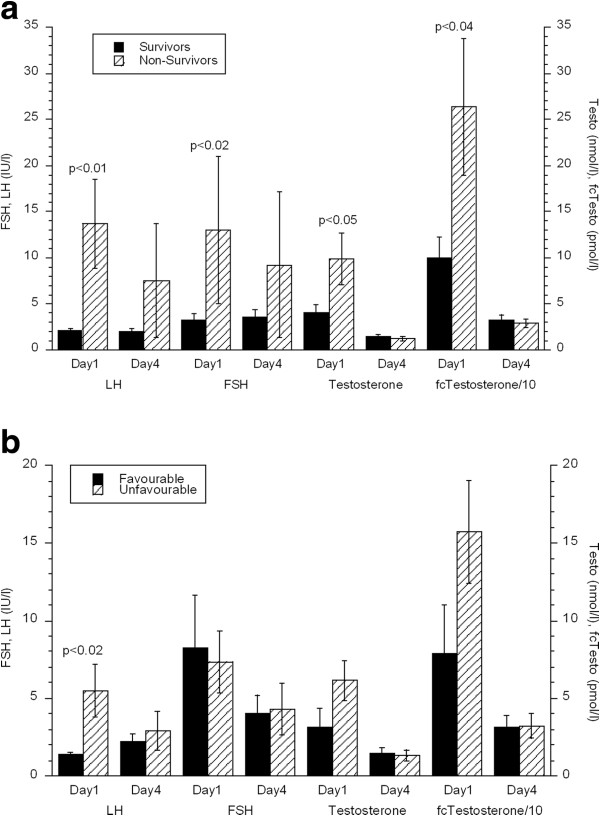
**a) Serum LH, FSH, testosterone and fc-testosterone levels day 1 and day 4 in male survivors and non-survivors 3 months after injury.** Note that the value for fc-testosterone is divided by 10. Wilcoxon sign rank test between groups. Values are means ± sem. **b**) Serum LH, FSH, testosterone and f fc-testosterone levels day 1 and day 4 in males with favorable (GOS 4-5) and unfavorable outcome 3 months after injury. Note that the value for fc-testosterone is divided by 10. Wilcoxon sign rank test between groups. Values are means ± sem.

LH levels were low in the acute phase after sTBI and LH below normal values were found in 16/29 male patients (55.2%) at day 1 and 17/29 (58.6%) day 4 after sTBI (Table 1). LH above normal was found in 2/29 (6.9%) patients both on day 1 and day 4 (Figure 1). LH day 1 was higher (13.7 ± 4.8 IU/L) in subjects deceased at 3 months as compared to survivors (2.1 ± 0.3 IU/L, p < 0.01) (Figure 3). Similar results were found for the unfavorable (5.5 ± 1.7 IU/L) vs. favorable group (1.4 ± 0.1 IU/L, p < 0.02) (Figure 3b). Accordingly, there was a negative correlation between day 1 LH levels and GOS at 3 months (ρ = −0.53, p < 0.004). Furthermore, levels of LH day 1 were significantly correlated to ICP_max_ (r = 0.49, p < 0.01) and to Marshall grade (ρ = 0.44, p < 0.02). There was no correlation between the LH levels and GCS, ISS and CPP_min_.

As for LH the level of FSH in the male patients was low in the acute phase after sTBI and decreased further from day 1 to day 4 (p < 0.003) (Table 1). FSH levels below normal were found in 3/29 (10.3%) at day 1 and in 11/29 (37.9%) at day 4, whereas FSH was above normal in 2/29 (6.9%) day 1 and 1/29 (3.4%) day 4 after sTBI (Figure 1). Day 1 FSH levels were higher in patients deceased at 3 months (13.0 ± 8.0 IU/L) as compared to survivors (3.3 ± 0.6 IU/L, p < 0.02). This difference was not found between the favorable and unfavorable groups. GOS at 3 months was negatively correlated with FSH day 1 (ρ = −0.46, p < 0.02) and as for LH there was a positive correlation between day 1 FSH and Marshall grade (ρ = 0.39, p < 0.04). There was no correlation between FSH and GCS, ISS, ICP_max_ and CPP_min_.

To investigate the possibility that the low testosterone was secondary to high prolactin we searched possible correlations between prolactin and LH, FSH, total testosterone and f-testosterone. No such correlation was found suggesting that the hypogonadotropic hypogonadism in acute TBI is not due to an inhibitory effect of elevated prolactin.

### Pituitary – gonadal axis in women

We were unable to establish in which phase in the menstrual cycle these severely injured women were. Neither could we for all subjects be certain whether they were pre- or postmenopausal or if they were on exogenous estrogens. Thus, these hormone levels could not be accurately interpreted and therefore not presented. However, LH, FSH and estradiol levels in woman were generally low.

### Somatotropic axis

There was a great variability in GH levels at all measured time points ranging from 0.2 ug/L to 99 ug/L. Mean GH levels showed no significant diurnal variation between morning and evening. No correlations were found between GH and GCS, ISS, Marshall grade, ICP_max_, CPP_min_ or GOS. There was a trend towards lower GH levels in deceased as compared to alive patients at 3 months, see Table 2.

**Table 2:** GH levels day 1 and day 4 in patients deceased and alive patients at 3 months post trauma

**S-GH (mIU/L)**	**Deceased (3 months)**	**Alive (3 months)**
Day 1 morning	11.9 ± 7.5	16.4 ± 2.6
Day 1 evening	10.6 ± 2.9	20.1 ± 3.3
Day 4 morning	8.9 ± 2.8	16.1 ± 3.0
Day 4 evening	8.6 ± 2.8	13.5 ± 2.1

Values are means ± sem.

There was a transient decrease in IGF-1, with low levels at day 1, which were restored towards normal at day 4 after sTBI, see Table 1. Thus, day 1 after sTBI 13/43 (30.2%) had levels below the age-related normal value, whereas none had high levels. Day 4, the corresponding figures were 1/44 (2.3%) and 3/44 (7.0%) respectively, Figure 1. Correspondingly, mean IGF-1 was significantly lower day 1 after sTBI than at day 4 (p < 0.0001) (Table 1). No statistically significant difference in IGF-1 levels between deceased and alive or unfavorable and favorable outcome at 3 months was observed. No correlation was found between IGF-1 and GCS, ISS, Marshall grade, ICP_max_, CPP_min_ or GOS.

### Outcome predictors

**Figure 4:**
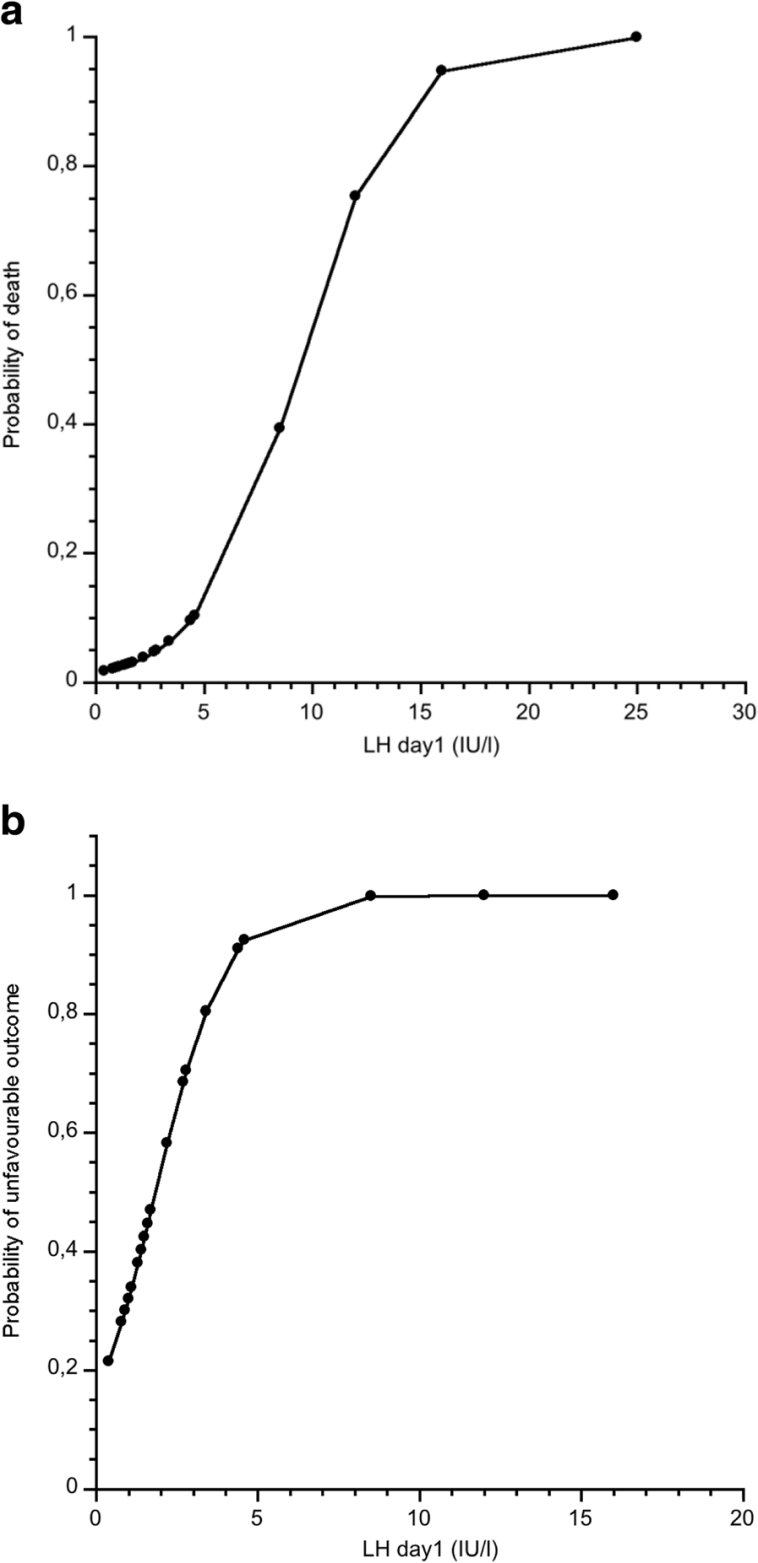
**a) Probability of death at 3 months related to serum LH levels day 1 after sTBI.** Probability results are from ROC analysis. **b**) Probability of unfavorable outcome at 3 months related to serum LH levels day 1 after sTBI. Probability results are from ROC analysis.

Among the hormones analyzed in the acute phase fT3 and TSH were most strongly and positively associated with outcome. In the male group LH and FSH was negatively associated with outcome. We attempted to evaluate the use of these hormones to predict clinical outcome. A logistic regression model with fT3 and TSH day 4 as independent factors and unfavorable/favorable outcome as dependent factor showed that the model significantly predicted (p < 0.03) outcome and fT3 contributed most to the prediction. Using GOS or dead/alive as dependent factors did not show any statistically significant prediction. When ICP_max_ was used in combination with fT3 and TSH day 1 in the whole group, the prediction of GOS at 3 months was p < 0.01 and ICP_max_ had the strongest effect on the model. When the results were dichotomized into dead or alive at 3 months and using the same independent factors a statistically significant predictive value (p < 0.01) was found and again ICP_max_ contributed most. Other combinations of hormone levels in the whole study group did not contribute to the outcome prediction. However, in men there was a clear prognostic value of LH and FSH. Thus GOS at 3 months was significantly predicted by day 1 LH in combination with FSH (p < 0.001) and LH contributed most to the prediction. Similar results were found in the prediction of dead/alive (p < 0.002) and unfavorable/favorable outcome (p < 0.01). When ICP_max_ was used in combination with day 1 LH and FSH, the prediction of GOS at 3 months was p < 0.005), dean/alive (p < 0.005) and unfavourable/favourable (p < 0.02) and in all predictions LH had the strongest effect on the model. In evaluating the power of LH day 1 to predict non-survivors/survivors in men a ROC analysis showed an AUC of 0.915 (p < 0.001) and the highest accuracy was at an LH level of 12.0 (IU/L), sensitivity 0.750, specificity 1.0. For a probability plot of outcome, see Figure 4a. Using the dicotomization of unfavorable/favorable outcome and LH day 1 resulted in an AUC of 0.774 (p < 0.003) and the highest accuracy at a LH level of 1.7 (IU/L), with a sensitivity of 0.687 and a specificity of 0.8462, see Figure 4b for a probability plot of outcome.

## Discussion

In this prospective study we describe alterations in pituitary-dependent hormone levels in the acute phase of sTBI, in a well-defined patient group treated according to an ICP-targeted therapy in a protocol-guided manner. The main findings of this study were: 1) A large proportion of the patients had low s-cortisol levels, below proposed cut-off levels for critical illness related corticosteroid insufficiency (CIRCI). Low s-cortisol levels (<276 nmol/L = 10 ug/dL) and even <100 nmol/L was not associated with higher mortality or unfavorable outcome at 3 months; 2) There was a significant association between early (day 1) strong suppression of the pituitary-gonadal axis and better survival and favorable outcome 3 months after sTBI in men. 3) Survival and favorable outcome (GOS 4–5) was associated with significantly higher levels of fT3 and TSH day 4 after sTBI. 4) In general there was no correlation between GCS, ISS, ICP_max_, CPP_min_ and hormone levels. Only in men day 1 LH was correlated to ICP_max_.

In the last decade several studies have shown permanent pituitary insufficiency after TBI to be considerably more common than previously recognized [3,8]. Therefore, screening to find patients and replace persistent hormone deficiency has been proposed and implemented in clinical practice at many centers [21]. The value of measure hormone levels in the acute phase after TBI is less clear, except when suspecting an acute cortisol insufficiency [8]. It is well known that the levels of most hormones are dramatically altered in acute critical illness as compared with resting baseline levels. Thus, interpreting hormone levels in the acute phase after TBI is complex and has to be related to the hormonal changes in any acute illness [22,23]. The potential benefits of replacement therapy of endocrine dysfunction in acute TBI are not known, except for the life-saving replacement of an acute ACTH-cortisol deficiency [8]. The few intervention studies aiming to replace (or over-replace) hormone deficiencies in acute critical illness have generally failed to show benefits of hormone treatment [23]. In contrast, there is evidence that pharmacological doses of glucocorticoids in TBI patients without cortisol deficiency increase mortality [24] and this has also been shown in a study using high doses of recombinant growth hormone in acute critical illness [25]. Thus, it is likely that most of the hormonal changes in acute critical illness can be attributed to physiological adaptation to severe physiological stress [8].

Most previous studies on hormone levels after TBI are on mixed materials, including mild, moderate and severe TBI and report varying rates of acute hormonal dysfunction [5,26,27]. The present study is the first study which prospectively studies hormone levels in the acute phase of severe TBI in a homogenous group of patients, strictly protocol-treated according to an ICP-targeted therapy based on the Lund concept [28]. We found profound changes for most pituitary dependent hormones in the acute phase after sTBI, i.e. low levels of thyroid hormones, strong suppression of the pituitary-gonadal axis and increased levels of prolactin, much in line with previous studies on acute TBI and other acute critical illness.

We have previously shown that our protocol-guided ICP targeted treatment seems to protect patients with severe TBI from clinical and subclinical seizures and thus reduces the risk of secondary brain injury and a raise in prolactin due to seizures [29].

Prostacyclin has been proposed to have beneficial effects in traumatic injury [30]. However, in a larger set of TBI patients these results were not confirmed [16]. Interestingly, it has been shown that prostacyclin in some situations influences pituitary hormone release [31]. Both plasma cortisol and prolactin increased after prostacyclin infusion. This effect has been suggested to be due to stress during prostacyclin infusion. However, the mechanisms are not fully understood [32]. We were unable to show any significant effect of prostacyclin upon hormone levels after acute sTBI. However, it is likely that the effect of the TBI upon hormone levels were so pronounced that the potential effect of prostacyclin was obscured.

### Hypothalamic-pituitary-adrenal axis

Elevated cortisol levels is a physiological response to critical illness to modulate metabolism to ensure energy substrates for vital organs, exert supporting effects on the circulatory system and suppress excessive immune system activation [22,33,34]. The primary endocrine task in acute TBI is not to overlook a clinically relevant deficiency of the hypothalamic-pituitary-adrenal (HPA) axis with acute cortisol insufficiency, a condition with life-threatening hyponatremia and hypotension, in need for prompt diagnosis and treatment with stress-doses of glucocorticoids. It should however be recognized that correct diagnosis of cortisol insufficiency in a critically ill patient is difficult and strict diagnostic criteria are still missing. Recent recommendations are to consider morning serum cortisol levels <300 nmol/L as highly suggestive of acute adrenal insufficiency and treat with stress-doses of i.v. hydrocortisone (i.e. 200–300 mg/d) [8]. We found low s-cortisol levels in more than 50% of the sTBI patients in our study. No patients in the current study were given glucocorticoids. In spite of this we found no association between low cortisol levels and increased mortality or unfavorable outcome. This is opposed to the Dublin group who found increased mortality in the patients with the lowest s-cortisol [8]. Contrary, in this study patients deceased at 3 months tended to have higher serum cortisol in the acute phase compared with survivors. This is in line with previous studies of cortisol levels in different types of critical illness [35] and may be attributed to a more severe critical illness and correspondingly higher stress response. Abnormalities in cortisol dynamics after severe traumatic brain injury are inconsistent, and correlations between serum cortisol levels and clinical outcomes have been conflicting since both high and low serum cortisol levels have been associated with poorer outcomes [36]. The CRASH-study reported increased mortality in TBI-patients treated with pharmacological doses of methylprednisolone [37]. Furthermore, experimental evidence suggests that suppressing elevated cortisol levels reduce neuronal damage after different insults [38]. On the other hand, in a recent study treatment with stress doses of hydrocortisone (200 mg/d) decreased the risk of hospital-acquired pneumonia and shortened mechanical ventilation in patients with sTBI and inadequate adrenal function, defined as basal s-cortisol <15 μg/dL (414 nmol/L) or rise in s-cortisol <9 μg/dL (248 nmol/L) after cosyntropin stimulation [39]. In that study a large proportion of the patients were treated with Etomidate and it has been criticized for difficulty to objective outcome parameters, since glucocorticoid therapy may blunt fever response, which was a criteria for pneumonia. Thus, more studies are needed to answer the question when a TBI-patient will benefit or not from replacement therapy with stress doses of glucocorticoids. In studies like this it is important to acknowledge the vast range of factors potentially influencing hormone levels in critically ill patients under full neuro-intensive care. In this study the patients have been treated in a strictly protocol-guided manner and most of the patients received the same drugs during the neuro-intensive care. Etomidate, which is a strong inhibitor of adrenal steroid hormone synthesis [40] was not used in any of the patients. Even so, some degree of confounding effects of e.g. anesthetics upon hormone levels cannot be excluded. Little is known about the effects of anesthetic drugs upon hormone levels in patients with sTBI. The effect of propofol, which was used in some of the patients is less clear. Some studies demonstrate a drop in cortisol levels during propofol infusion [41,42], whereas others show no effect on hormone levels [43]. More studies on the isolated effect of anesthetic drugs upon hormone levels are needed, since these cannot be avoided during the early treatment of patients with severe traumatic head injury.

### Thyroid axis

Levels of fT4 and fT3 were low in the acute phase of sTBI, with a significant decrease from day 1 and 4, in line with a previous study [44]. Interestingly, fT3 levels were significantly lower at day 4 in patients who died within 3 months after sTBI compared to survivors. Furthermore, survivors with an unfavorable outcome at 3 months had significantly lower fT3 levels compared to patients with favorable outcome. Previous studies have suggested an association between stronger suppression of the hypothalamic-thyroid axis in more severe injuries and poor outcomes [44,45]. In line with this we found a negative correlation between day 4 TSH-levels and Marshall CT grade score, i.e. worse radiological findings were associated with lower s-TSH day 4 after sTBI. The observed decreases in serum concentration of both thyroid hormones and TSH are consistent with a central suppression of the hypothalamic-pituitary-thyroid (HPT) axis. This is supported by post mortem studies showing a decreased expression of thyrotropin-releasing hormone in the hypothalamic paraventricular nucleus of patients with a decreased serum T3 level [46]. In critical illness, serum T3 may even become unmeasurable without giving rise to an elevated concentration of serum TSH. It is at present not clearly established whether this reflects an adaptation of the organism to illness or instead a potentially deleterious condition leading to hypothyroidism at tissue level. It is likely that the transient down regulation at all levels of the HPT axis (decreased TRH and TSH at the hypothalamic-pituitary level, and a decreased T3 due to altered peripheral deiodinase activity) is part of the neuro-endocrine adaptation to critical illness in an attempt to save energy. In this study of only severe TBI, we found that a more pronounced and prolonged suppression of TSH was associated with unfavorable outcome, and more severe CT-findings suggesting that a less pronounced and prolonged central down regulation of the HPT-axis may be a marker of a less severe TBI and/or a stronger capacity to adapt and regain hypothalamic-pituitary function after TBI, with a more normal TSH-response (i.e. higher) to low thyroid hormone levels.

### Prolactin

We found elevated levels of serum prolactin, with an increase from day 1 to day 4 after TBI. Our results are in accordance with previous studies showing hyperprolactinemia in more than 50% of patients in the early, acute phase post-TBI [26,47], established in ranges from mild to severe traumatic head injury [5]. Day 1 serum prolactin levels were significantly negatively correlated with CPP_min_ and positively correlated to ICP_max_. Apart from this we found no correlation between serum prolactin levels day 1 or day 4 and severity of TBI, which was reported in some previous studies [26,45,48]. Nor could prolactin levels in the acute phase be used as a prognostic factor of clinical outcome. Gonadotropin (LH/FSH) and testosterone secretion is inhibited by elevated levels of prolactin, e.g. in patients with prolactinomas. However, in this study the elevated prolactin levels in acute TBI did not appear to contribute to the suppression of the pituitary-gonadal axis, which is in line with previous studies [49].

### Pituitary – gonadal axis

Central inhibition of the pituitary-gonadal axis is a consistent finding in critical illness, including TBI and considered an adaptive response to severe physiological stress, i.e. an appropriate temporary swich-off of anabolic androgens in circumstances of acute stress, to preserve energy and metabolic substrates for vital functions. In line with previous studies we found strong central inhibition of the pituitary-gonadal axis already at day 1, with further suppression at day 4, when 100% of the male patients showed testosterone levels below reference range. Furthermore, almost all of the men had low levels of LH and FSH both at day 1 and 4. This is in agreement with a previous study showing a high incidence of hypogonadotropic hypogonadism in the immediate post-TBI period [50]. Previous studies have clearly demonstrated that testosterone levels in males and estrogen levels in females significantly fall within the first 24 hours following TBI and remain low for 7–10 days [26,50].

Novel findings in this study were significant correlations between lower levels of LH and FSH day 1 and worse brain injury according to the Marshall CT grade score. This is in line with previous reports on associations between more severe TBI (often lower GCS) and stronger suppression of the hypothalamus-pituitary-gonad-axis [8]. Interestingly, male patients alive at 3 months after TBI had significantly lower LH, FSH and testosterone levels day 1 vs. non-survivors. Day 1 serum LH was also lower in male patients with a favorable outcome 3 months after TBI vs. unfavorable outcome, i.e. GOS 1–3. These findings in this study suggest that very severe brain injury may hamper the adaptive, physiological suppression of the pituitary-gonadal axis and this inability is a poor prognostic sign.

### Somatotropic axis

Growth hormone is normally released in a highly pulsatile manner. Therefore analyses regarding sub- and supra-normal levels may be misleading. We found a great variability in GH levels at all measured time points. Mean serum GH levels showed no significant diurnal variation, i.e. between morning and evening. Interestingly, there was a trend towards higher serum GH in patients who survived 3 months than in those who succumbed to their injuries. We also found a transient decrease in serum IGF-1 with low levels at day 1, which were restored towards normal at day 4 after sTBI. Low IGF-1 with elevated GH levels have been shown in the acute post-traumatic phase, as well as a normalization of GH and increase of IGF-1 in the following weeks after trauma [51]. This has been attributed to a state of acquired peripheral GH resistance in critical illness [52]. Contradictory literature is available on the GH levels following severe traumatic brain injury as reported by various authors. Chiolero et al. found elevated GH levels in the acute phase [45]. Hackl et al. reported elevated GH levels in patients with high ICP [53]. Della Corte et al. showed relatively normal GH levels in the acute phase, and improving levels of IGF-1 1 and 2 weeks after trauma compared to 2 days after trauma [54]. In a more recent study, in which mild, moderate and severe injuries were mixed the GH levels overall remained relatively normal or slightly elevated throughout the acute setting [50]. Growth hormone is normally released in a highly pulsatile manner. Furthemore, clonidine and metoprolol have been shown to increase GH sekretion. [55,56] Therefore analyses of GH sampled only twice daily under multiple pharmacological treatment should be interpreted with caution.

### Prediction of outcome

In the search for biomarkers of clinical outcome after sTBI we tested the hypothesis that hormone levels may predict outcome at 3 months post-injury. Day 4 fT3 and TSH levels were shown to be predictive factors for outcome. Interestingly, in men day 1 LH and FSH were predictive factors of outcome and LH was the main predicting factor. However, by combining the above-mentioned hormone levels with ICP_max_ it was clear that ICP_max_ still is the main predicting factor of outcome in patients treated at our department. By using LH day 1 in men the ROC analysis showed a high AUC and a rather good sensitivity and a good specificity. The probability of worse outcome increased with increasing levels of LH day 1.

### Study limitations

This study is limited by the fact that GH, LH and FSH hormone levels were assessed only once daily. Given the known pulsatile secretory pattern of GH, LH and FSH, the true pattern of their release in the acute setting would require repeated sampling at close intervals. Also, due to lack of information about the females menstrual status the female gonadal axis could not be analyzed in detail. In this study no hormonal stimulation tests were performed, which in some cases may provide more detailed information about responsiveness of the hypothalamic-pituitary axis. Although such stimulation tests were done in some prior studies, they are challenging to do during the first days after the trauma with the patient under full neuro-intensive care. Also, the intention of the study was not to explore the patency of the hypothalamic-pituitary axis but to investigate the trauma induced effects on hormonal levels.

Many of the patients (i.e. 69%) in the study suffered from multiple injuries [57]. The data does not allow discrimination of what proportion of the hormone alteration is caused by the TBI itself and how much is caused by the extracranial injuries/critical illness situation. Furthermore, according to the treatment protocol most of the patients received blood transfusions. No analyses are made regarding a possible association between blood transfusions and hormone levels [58].

The clinical impact of the acute alterations of hormone levels in patients with severe brain injury is still largely unknown. Most changes are likely to represent an adaptive physiological response to critical illness. However, experimental studies indicate that hormonal effects upon the CNS in acute, sub-acute and long-term recovery phases may be of importance for secondary neuronal damage and plasticity/recovery processes. In this study we find that hormone levels in the early acute phase of sTBI may be used in models to predict clinical outcome. However, traditional acute phase variables, i.e. ICP appear superior and large cohorts are needed for further evaluation and confirmation of reliable screening markers. Future studies should be designed to ensure a high diagnostic robustness for proper identification of reliable predictors, as the results may be highly dependent on diagnostic pitfalls.

In conclusion, we found pronounced dynamic alterations in pituitary-dependent hormone levels day 1 and 4 after severe TBI. Cortisol levels below proposed cut-off limits for adrenal insufficiency were found in a large proportion of the patients without association to unfavorable clinical outcome. Stronger early suppression of the pituitary-gonadal axis was associated with a more favorable outcome whereas a prolonged suppression of the thyroid axis was associated with higher mortality and poor functional outcome. Whether these findings are applicable for other groups of patients with sTBI treated by other treatment regimes has to be further evaluated.

## Competing interests

The authors declare that they have no competing interests.

## Authors’ contributions

ZO: Carried out surgery on some of the patients, collected data and went through patient charts, wrote the manuscript, analysis and interpretation of data, revising the manuscript critically. PD: analysis and interpretation of data, revising the manuscript critically, wrote some parts of the manuscript. LOK: Designed the study, collected data, analysis and interpretation of data, revising the manuscript critically. All authors read and approved the final manuscript.
